# Immunometabolic regulation of cardiac macrophages in heart failure with preserved ejection fraction

**DOI:** 10.3389/fimmu.2026.1896936

**Published:** 2026-09-07

**Authors:** Jiayun Liang, Manman Su, Renjie Liu, Tianlang Li, Ziyan Qin, Yi Wang

**Affiliations:** Department of Regenerative Medicine, School of Pharmaceutical Sciences, Jilin University, Changchun, China

**Keywords:** cardiac resident macrophages, heart failure with preserved ejection fraction, immunometabolism, inflammation, monocyte-derived macrophages

## Abstract

Heart failure with preserved ejection fraction (HFpEF) is a complex syndrome driven by comorbidities, chronic low-grade inflammation, and cardiac remodeling. Cardiac resident macrophages (CRMs) and monocyte-derived macrophages (MDMs) play central roles in linking immunometabolism to HFpEF pathogenesis. Resident CCR2^-^ macrophages maintain homeostasis and tissue repair, whereas monocyte-derived CCR2^+^ macrophages drive inflammation, fibrosis, and diastolic dysfunction. In HFpEF, comorbidity-induced metabolic shifts promote pro-inflammatory macrophage polarization, while metabolites like fatty acids, lactate, and succinate modulate these responses through metabolic and epigenetic pathways. Targeting specific macrophage subsets offers promising therapeutic avenues, though challenges like off-target effects and clinical translation remain. This review summarizes cardiac macrophage heterogeneity, immunometabolic crosstalk, and regulatory mechanisms in HFpEF, highlighting emerging subset-specific therapeutic strategies.

## Graphical Abstract

**Figure:**
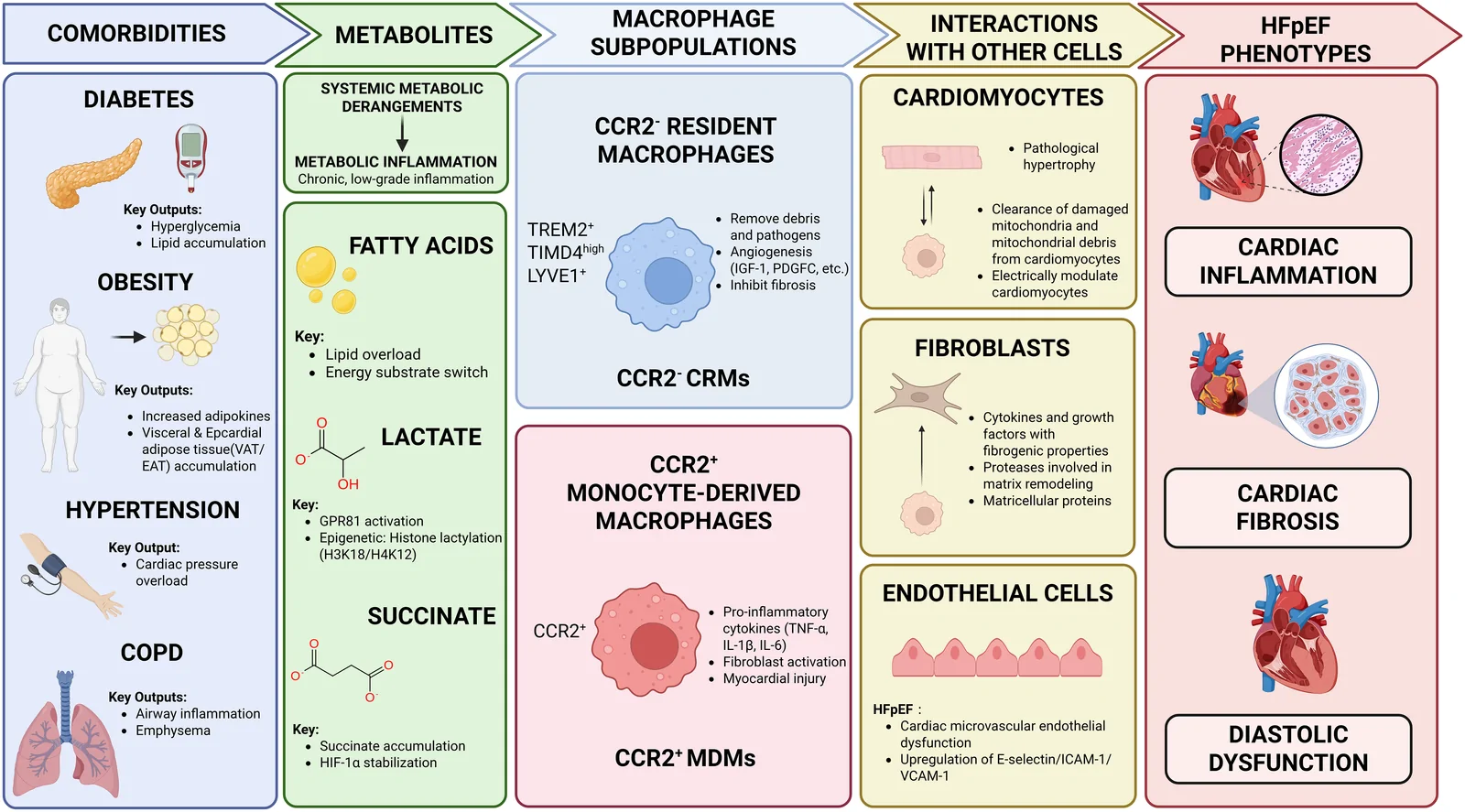
Comorbidities associated with HFpEF—including diabetes, obesity, hypertension, and COPD—induce metabolic changes and chronic inflammation. Alterations in metabolic byproducts such as fatty acids, lactate, and succinate regulate the activation and polarization of cardiac macrophages. Interactions between macrophages and cardiomyocytes, fibroblasts, and endothelial cells exacerbate cardiac inflammation, remodeling, and diastolic dysfunction, thereby driving the progression of HFpEF. HFpEF, heart failure with preserved ejection fraction; VAT, visceral adipose tissue; EAT, epicardial adipose tissue; COPD, chronic obstructive pulmonary disease; GPR81, G protein-coupled receptor 81; HIF-1α, hypoxia-inducible factor 1α; CCR2, C-C chemokine receptor 2; CRMs, cardiac resident macrophages; MDMs, monocyte-derived macrophages; TREM2, triggering receptor expressed on myeloid cells 2; TIMD4, T-cell immunoglobulin and mucin domain-containing protein 4; LYVE1, lymphatic vessel endothelial hyaluronan receptor 1; IGF-1, insulin-like growth factor 1; PDGFC, platelet-derived growth factor C; TNF-α, tumor necrosis factor-α; IL-1β, interleukin-1β; IL-6, interleukin-6; ICAM-1, intercellular adhesion molecule-1; VCAM-1, vascular cell adhesion molecule-1.

## 1 Introduction

Heart failure (HF) is a clinical syndrome in which symptoms or signs emerge from structural or functional abnormalities of the heart (1). Affecting over 26 million individuals globally, HF has emerged as a mounting public health concern (2). Using left ventricular ejection fraction (LVEF) as a criterion, HF can be divided into three subtypes: heart failure with reduced ejection fraction (HFrEF), commonly defined as LVEF ≤40%; heart failure with mildly reduced ejection fraction (HFmrEF), where LVEF falls between 41% and 49%; and heart failure with preserved ejection fraction (HFpEF), characterized by LVEF greater than 50% (1, 3). Among the various HF subtypes, HFpEF in particular has seen a continued increase in incidence, and its prevalence is anticipated to surpass that of HFrEF in the near term (4). HFpEF is a complex syndrome closely linked to many non-cardiac comorbidities (5), such as hypertension (6), obesity (7), diabetes mellitus (8) and chronic obstructive pulmonary disease. These comorbidities collectively promote systemic immune cell activation and pro-inflammatory cytokine release, thereby triggering and maintaining a state of inflammation in the heart, ultimately leading to myocardial remodeling and dysfunction (9). Emerging evidence indicates that the interaction—both direct and indirect—between metabolic processes and inflammatory immune cells promotes cardiac inflammation as well as fibrosis. Additionally, the buildup of immune cells inside the heart, driven by a perturbed metabolome, further exacerbates these pathological changes. Together, these mechanisms contribute to the pathogenesis of HFpEF (10).

Within the heart, macrophages constitute a predominant cell population (11), and act as critical regulators of both cardiac health and disease states (12). As phagocytic immune cells, they fulfill multiple functions in preserving tissue homeostasis and responding to tissue injury. Nevertheless, they also participate in myocardial fibrosis and the development of cardiac diastolic dysfunction (13). As research progresses, new evidence has been obtained for the role of macrophages in immune metabolism and the regulation of inflammatory pathways by nutrients and metabolites (14). The relationship between macrophage abundance/function and disease progression in human HF supports the development of therapeutics that inhibit macrophage recruitment and neutralize damaging inflammatory functions of macrophages to promote recovery of the failing heart (15). Here, we explore the immunometabolic regulatory mechanisms of macrophages in HFpEF pathogenesis and review novel macrophage-targeted therapeutic approaches.

Scope of this review

This review aims to provide a comprehensive analysis of the immunometabolic regulatory mechanisms governing cardiac macrophages in the context of HFpEF (Graphical Abstract). Specifically, this article seeks to answer the following questions:

1. How cardiac macrophage subsets, specifically CCR2^-^ versus CCR2^+^ populations, undergo distinct metabolic reprogramming and functional polarization during the pathogenesis of HFpEF.
2. How comorbidity-triggered systemic metabolic stress integrates with local cardiac macrophage responses, with a particular emphasis on the cardiopulmonary continuum and systemic-to-cardiac immune signaling.
3. How established and emerging therapeutic strategies target these macrophage-specific immunometabolic pathways, and what major obstacles exist in subset-specific targeting and clinical translation.

Unlike previous reviews that have addressed general inflammatory processes in heart failure, this review offers a more detailed focus on the macrophage-centered immunometabolism within the HFpEF heart, incorporating the latest findings from single-cell transcriptomic data to characterize macrophage heterogeneity and their unique regulatory crosstalk with cardiomyocytes, fibroblasts, and endothelial cells.

## 2 Cardiac macrophage subpopulations

Macrophages exhibit heterogeneity (16). Traditionally, macrophages are classified into two phenotypes, namely pro-inflammatory M1 and anti-inflammatory/reparative M2, based on surface markers, functional characteristics, and cytokine production (17). M1 macrophages primarily secrete pro-inflammatory mediators to promote inflammatory progression, mediate reactive oxygen species (ROS)-induced tissue damage, and impair tissue regeneration along with wound healing (2). Macrophages of the M2 type are known as alternatively activated or anti-inflammatory macrophages. They stimulate tissue repair and regeneration while also exerting pro-angiogenic effects (18). Depending on the stimuli they receive, M2 macrophages can be classified into four functionally distinct subtypes: M2a, M2b, M2c, and M2d (19). M2a macrophages promote tissue repair and fibrosis by secreting transforming growth factor-β (TGF-β) and extracellular matrix components. M2b macrophages exert an immunomodulatory effect through high secretion of interleukin-10 (IL-10), while simultaneously maintaining secretion of the pro-inflammatory factor tumor necrosis factor-α (TNF-α), thereby exhibiting a “mixed phenotype”. M2c macrophages are highly specialized in phagocytosis, tissue remodeling, and anti-inflammation. M2d macrophages primarily promote angiogenesis by secreting vascular endothelial growth factor (20). However, due to the significant plasticity exhibited by macrophages and the dynamic expression of cell surface markers (21), the M1-M2 classification systems and definitions are increasingly recognized as imperfect. Recent single-cell RNA sequencing (scRNA-seq) technologies have revolutionized our understanding, revealing that the mammalian heart harbors multiple functionally distinct, highly specialized cardiac macrophage clusters that extend far beyond the classic binary M1/M2 paradigms. These transcriptomically defined subsets, such as T-cell immunoglobulin and mucin domain-containing protein 4 (TIMD4)^+^ resident clusters, triggering receptor expressed on myeloid cells 2 (TREM2)^+^ resident populations, and major histocompatibility complex class II (MHC-II)^high^ antigen-presenting cells, exhibit unique regulatory profiles during cardiac homeostasis and pathological remodeling (22). Based on cellular origin and tissue residency status, cardiac macrophages (CMs) are classified into two primary categories: cardiac resident macrophages (CRMs) and monocyte-derived macrophages (MDMs) (23). C-C chemokine receptor 2 (CCR2) has been shown to be a surface marker distinguishing the two cell types, with CRMs lacking CCR2 expression on their surface while MDMs express CCR2 (24). Similar to M1/M2 macrophages, these two macrophage types play different roles in inflammation after cardiac injury. Monocyte-derived macrophages exhibit a pro-inflammatory profile and lack reparative functions, whereas cardiac-resident macrophages have anti-inflammatory functions and facilitate cardiac recovery through cardiomyocyte proliferation and angiogenesis (25). One study has described three novel macrophage subgroups in the adult murine myocardium: CCR2^-^MHC-II^low^, CCR2^-^MHC-II^high^, and monocyte-derived CCR2^+^MHC-II^high^ (23). This classification system is supported by single-cell RNA sequencing data (22) and lays the groundwork for subsequent discussions on the recruitment of monocytes and monocyte-derived macrophages to the injured heart.

### 2.1 Cardiac resident macrophages

**Figure 1:**
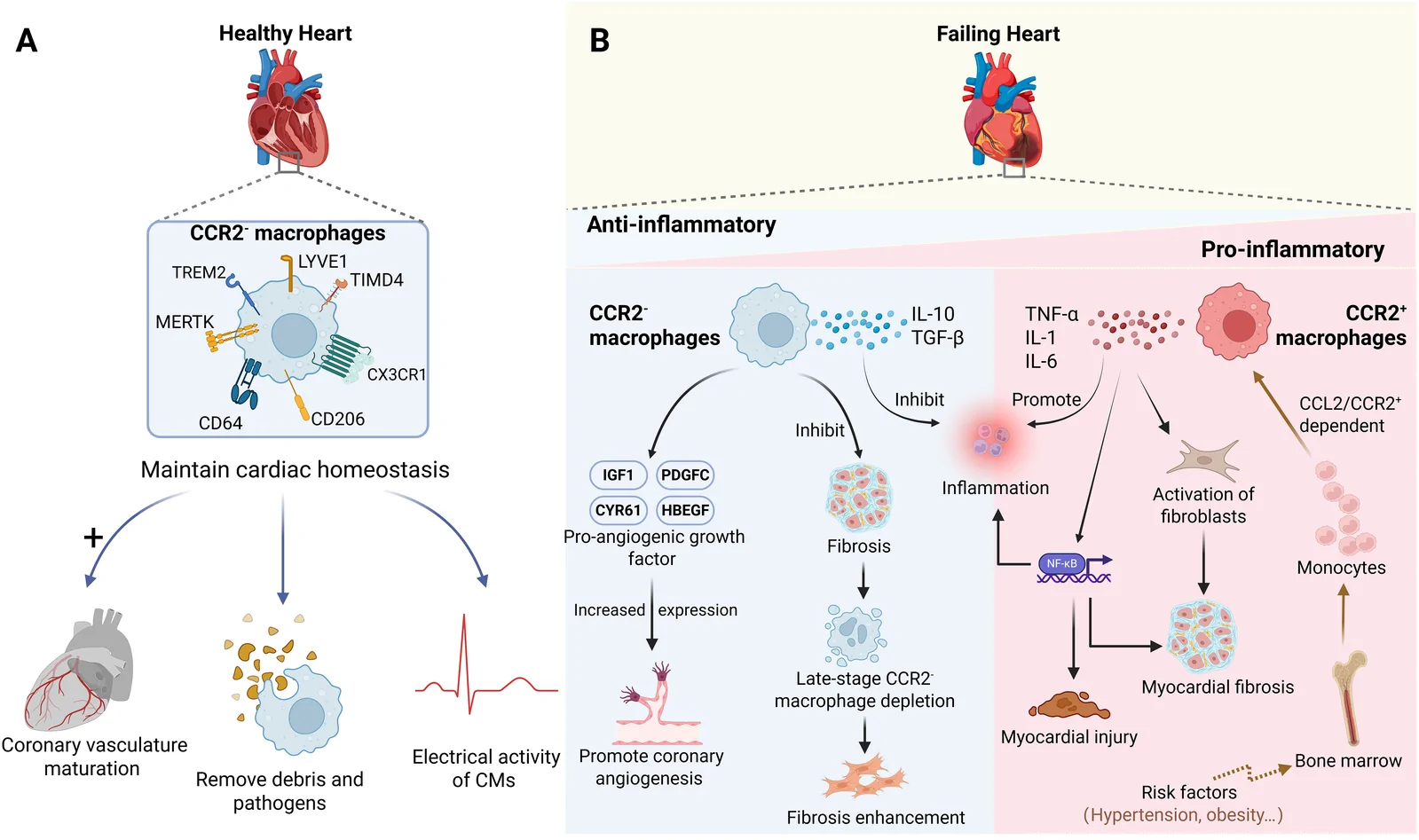
Classification and functions of cardiac macrophages. **(A)** Representative surface markers of CCR2^-^ macrophages and CCR2^-^ macrophages’ functions in a healthy heart. In a healthy heart, CCR2^-^ macrophages primarily maintain cardiac homeostasis by clearing debris and pathogens, promoting the maturation of coronary artery tissue and participating in cardiac electrophysiology. **(B)** The roles of CCR2^-^ macrophages and CCR2^+^ macrophages in a failing heart. CCR2^-^ macrophages promote coronary angiogenesis, exert anti-inflammatory effects, and inhibit fibrosis. Monocyte-derived CCR2^+^ macrophages release pro-inflammatory factors, promote fibroblast activation, and lead to myocardial injury. CCR2, C-C chemokine receptor 2; TREM2, triggering receptor expressed on myeloid cells 2; LYVE1, lymphatic vessel endothelial hyaluronan receptor 1; TIMD4, T-cell immunoglobulin and mucin domain-containing protein 4; MERTK, MER proto-oncogene, tyrosine kinase; CX3CR1, C-X3-C motif chemokine receptor 1; CD64, cluster of differentiation 64; CD206, cluster of differentiation 206; IL-10, interleukin-10; TGF-β, transforming growth factor-β; TNF-α, tumor necrosis factor-α; IL-1β, interleukin-1β; IL-6, interleukin-6; IGF1, insulin-like growth factor 1; PDGFC, platelet-derived growth factor C; CYR61, cysteine-rich angiogenic inducer 61; HBEGF, heparin-binding EGF-like growth factor.

In the healthy adult heart, cardiac resident macrophages make up 6% to 10% of the cells found in the myocardium (26). Research shows that these macrophages mainly display MHC-II^low^ and CCR2^-^. They also have characteristic marker gene profiles, such as *Timd4^+^*, lymphatic vessel endothelial hyaluronan receptor 1 (*Lyve1)^+^* and folate receptor beta (*FolR2)^+^*, among others (27). Cardiac resident macrophages originate from yolk sac progenitors and are mostly located inside the myocardial wall and near the coronary vasculature (28). In a healthy heart, the primary role of CRMs is to maintain cardiac homeostasis, such as promoting the progression and maturation of coronary arteries, participating in cardiac electrophysiology (25), and clearing debris and pathogens (29), as shown in **Figure 1A**. In response to external pathological stimuli, these resident macrophages proliferate to promote myocardial cell survival and physiological hypertrophy, inhibit detrimental monocyte recruitment, and stimulate vasodilation (24). CCR2^+^ macrophages function as central promoters of inflammation, monocyte recruitment, and adverse left ventricular remodeling. Conversely, in murine models of myocardial injury, resident CCR2^-^ macrophages suppress monocyte recruitment. When CCR2^-^ macrophages are depleted, monocyte recruitment increases (30). The mechanisms through which CCR2^-^ macrophages differentially modulate monocyte recruitment and subsequent fate determination remain incompletely understood. Possible mechanisms include releasing anti-inflammatory factors such as IL-10 and TGF-β, regulating the activation of CCR2^+^ macrophages, and directly signaling to recruited monocytes (30). Recent studies in murine pressure-overload models provide evidence that resident CCR2^-^ cardiac macrophages constitute a protective subset that facilitates adaptive remodeling and promotes cardiac survival under chronic heart failure conditions (31). Research in murine models has also shown that during cardiac pressure overload, CCR2^-^ macrophages play a key role in stimulating angiogenesis and inhibiting fibrosis (32). The mechanism promoting angiogenesis is linked to upregulated mRNA expression of the pro-angiogenic growth factors insulin-like growth factor 1 (IGF-1), platelet-derived growth factor C (PDGFC), cysteine-rich angiogenic inducer 61 (CYR61), and heparin-binding EGF-like growth factor (HBEGF) in CCR2^-^ macrophages (31), as shown in **Figure 1B**.

The classification of cardiac resident macrophages is primarily based on surface markers. Several inflammation-associated surface markers, including TREM2, C-X3-C motif chemokine receptor 1 (CX3CR1), LYVE1, TIMD4, MER proto-oncogene, tyrosine kinase (MERTK), cluster of differentiation 64 (CD64), and cluster of differentiation 206 (CD206), have been identified through single-cell RNA sequencing studies (33). Although the specific functions of many macrophage phenotypes and their markers remain poorly understood, certain phenotypes have been identified as possessing distinct immune functions. TREM2-expressing macrophages constitute a functional subset of CRMs (34). TREM2 is a single-transmembrane receptor expressed on monocytes and macrophages that recognizes anionic ligands, including lipoproteins, phospholipids, and bacterial products (35). Upon activation through ligand binding, TREM2 initiates an intracellular signaling cascade that enhances the clearance of apoptotic cells and metabolic waste (36), maintains tissue homeostasis and repair, and suppresses the production of pro-inflammatory factors, thereby promoting the resolution of inflammation (35). Recently, in a mouse model of HFpEF driven by hypertension, it was demonstrated that mice with genetic deletion of the *Trem2* gene exhibited more severe cardiac hypertrophy and diastolic dysfunction compared to wild-type control mice, indicating that TREM2^+^ macrophages are protective in cardiac remodeling (37). In murine studies, TIMD4 enables macrophages to recognize and eliminate apoptotic cells. The TIMD4^+^ resident cardiac macrophages are thought to favor oxidative phosphorylation, which in turn sustains their tissue-reparative and anti-inflammatory functions (27).

### 2.2 Monocyte-derived macrophages

CCR2^+^ macrophages are derived from bone marrow-derived monocytes, primarily reside within scarred or fibrotic areas, and contribute to inflammation, fibrosis, and ventricular remodeling. Their population is sustained through monocyte infiltration and proliferation (25). CCR2^+^ macrophages produce substantial amounts of interleukin-1β (IL-1β), interleukin-6 (IL-6) and TNF-α following lipopolysaccharide (LPS) stimulation or exposure to necrotic cardiomyocytes (24). These pro-inflammatory factors directly impair cardiomyocyte function while further activating other immune cells within the heart and triggering downstream inflammatory signaling pathways, such as nuclear factor κB (NF-κB) and the NOD-like receptor family pyrin domain-containing protein 3 (NLRP3) inflammasome (38), leading to inflammation, adverse cardiac remodeling, and the onset of heart failure (39), as shown in **Figure 1B**. When HFpEF develops, MDMs in the circulation and tissue increase, mainly driven by local macrophage proliferation and infiltration of monocytes derived from hematopoietic progenitor cells in the bone marrow and spleen (40). This is consistent with one of the characteristics of early HFpEF—circulating pro-inflammatory monocytosis and imbalance between monocyte-derived macrophages and resident macrophages. In murine HFpEF models, CCR2 knockout prevents CCR2^+^ macrophage infiltration, restores the proportion of resident macrophages, and significantly improves left ventricular hypertrophy (41). However, blocking CCR2 on macrophages is insufficient to improve diastolic dysfunction and fibrosis (42). This may be because left ventricular hypertrophy and myocardial fibrosis are two distinct pathological processes. Left ventricular hypertrophy is primarily driven by mechanical stress, which directly induces cardiomyocyte hypertrophy. In the early stages, CCR2^+^ macrophages amplify the hypertrophic response through pro-inflammatory factors; therefore, blocking their infiltration can significantly reduce left ventricular hypertrophy (43). In contrast, myocardial fibrosis centers on fibroblast activation and extracellular matrix deposition. Its onset and progression involve not only inflammation-related pro-fibrotic pathways but also direct activation of fibroblast signaling by a subset of C-C motif chemokine ligand 24 (CCL24)^+^ resident macrophages via the CCL24-CCR3 axis (44). Therefore, blocking the infiltration of CCR2^+^ macrophages alone is insufficient to eliminate the pathological process of myocardial fibrosis.

## 3 The effects of macrophages on the immunometabolic regulation of other cells

### 3.1 Macrophages and cardiomyocytes

Through single-cell transcriptomics analysis, cardiomyocytes have obvious transcriptional differences in HFpEF and are considered as the main cell type with obvious changes in HFpEF (45). In HFpEF, both cardiomyocytes and immune cells adjust their metabolic programs in response to inflammatory signals from the heart and systemic circulation. Soluble mediators and metabolites produced by immune cells and cardiomyocytes act as signaling molecules to trigger metabolic crosstalk between these two cells, thereby affecting the balance between inflammation resolution and adverse cardiac remodeling (46).

Left atrial enlargement and left ventricular concentric hypertrophy are cardiac structural abnormalities associated with HFpEF (47, 48). Findings suggest that, in response to external pathological stimuli, cardiac CCR2^-^ macrophages undergo proliferation, thereby promoting vasodilation and physiological hypertrophy, preventing deleterious monocyte recruitment, and stimulating vascular expansion (30). *In vitro* and in murine co-culture systems, CCR2^+^ macrophages secrete pro-inflammatory cytokines TNF-α, IL-1β, and IL-6, which activate MAP kinase and NF-κB signaling pathways and inhibit the Akt-mTOR pathway in neighboring cardiomyocytes, thereby promoting pathological hypertrophy of cardiomyocytes (49), and playing a critical role in left ventricular (LV) inflammation and remodeling (50). Therapies targeting CCR2^+^ macrophages, such as inhibiting pro-inflammatory cytokines like IL-1β (51), have shown promising results in preclinical models. A subset of cardiac resident macrophages with high expression of TREM2 has also been shown in murine HFpEF models to inhibit myocardial hypertrophy and suppress inflammation, exerting a protective role in cardiac remodeling (52). Thus, TREM2 emerges as an attractive therapeutic target for cardiovascular diseases (53).

In addition to myocardial hypertrophy, other crosstalk outcomes exist between macrophages and cardiomyocytes. Myocardial injury may play an important, previously underappreciated role in the pathophysiology of HFpEF (54). Regarding the maintenance of myocardial homeostasis, the cooperation between immune cells and parenchymal cells is key to keeping the heart healthy. In both steady-state healthy hearts and acute injury models, cardiac macrophages help maintain the metabolic homeostasis of cardiomyocytes and the normal pumping function of the heart by phagocytosing the decaying mitochondria released by cardiomyocytes (55). When cardiac macrophages are depleted or macrophage phagocytic receptors are lacking, dysfunctional mitochondria within cardiomyocytes increase, thereby reducing ATP production (56). These hearts also exhibit impaired cardiac filling similar to HFpEF. ScRNA-seq results indicate that in a mouse model of HFpEF, distinct macrophage subpopulations may directly affect cardiomyocyte function by activating several stress-related pathways (57). The TIMD4^+^ subset exhibited enrichment in nuclear receptor signaling pathways recognized for their anti-inflammatory characteristics (58), whereas MHC-II^high^ and CCR2^+^ macrophages displayed marked enrichment in pro-inflammatory pathways (59), reflecting the heterogeneity of cardiac macrophages. However, in addition to serving as a major driving force, macrophages also respond to metabolic signals originating from cardiomyocytes. Studies have shown that when cardiac pressure is overloaded, cardiomyocytes are the active initiators of the inflammatory response, while macrophages are the recruited executors. In the early stages of mechanical overload, Calcium/Calmodulin-Dependent Protein Kinase II δ (CaMKIIδ) is activated within cardiomyocytes, which in turn activates NF-κB and generates ROS, thereby activating the NLRP3 inflammasome in cardiomyocytes in response to hemodynamic stress (60). The inflammasome produces pro-inflammatory cytokines IL-1β and IL-18, which promote the infiltration of CCR2^+^ macrophages into the heart, amplifying and sustaining cardiac inflammatory responses (61). This indicates that cardiomyocytes serve as the primary site for inflammatory gene expression, challenging the notion that macrophages initiate and mediate myocardial inflammation (60).

Apart from being closely related to the inflammatory response, macrophages in the heart can also electrically modulate cardiomyocytes (22). CCR2^-^ resident macrophages in the atrioventricular node can electrically couple to cardiomyocytes via gap junctions containing connexin Cx43, connecting the cytoplasm of two adjacent cells to enable communication. The macrophages’ electrotonic load depolarizes resting cardiomyocytes, reduces their action potential upstroke velocity and overshoot, and aids early repolarization (22). This indicates that CCR2^-^ macrophages possess the function of modulating cardiomyocytes’ electrophysiological properties and improving atrioventricular nodal conduction.

### 3.2 Macrophages and fibroblasts

In human HFpEF, cardiac fibrosis represents a major axis of reparative and adverse remodeling (62). Macrophage–fibroblast interactions have a central role in cardiac fibrosis (63, 64). Activated macrophages can act as initiators of the fibrosis process and regulate fibrosis in the following three ways: by serving as a major source of cytokines and growth factors with fibrogenic properties; by secreting proteases involved in matrix remodeling; and by producing matricellular proteins (65). Different macrophage subsets may contribute to the fibrotic responses through the secretion of various mediators, including pro-inflammatory cytokines (such as TNF-α and IL-1β), anti-inflammatory (IL-10) and fibrogenic (TGF-β) cytokines, or growth factors, including IGF-1, fibroblast growth factors (FGFs), and platelet-derived growth factors (PDGFs) (65). CCR2^+^ macrophages regulate IL-1β through bromodomain-containing protein 4 (BRD4)-dependent chromatin remodeling, thereby autonomously activating fibroblasts (66). Chronic HF hearts contain the greatest proportion of fibroblast activation protein (FAP)/periostin (POSTN) fibroblasts (64). IL-1β produced by CCR2^+^ macrophages can directly signal to FAP/POSTN fibroblasts in murine models of chronic heart failure, driving fibroblast activation and exacerbating cardiac fibrosis. Correlative evidence in human HFpEF hearts supports the relevance of this pathway (64). IL-10 contributes to a macrophage phenotype shift toward a profibrotic subset, which activates fibroblasts. FACS-purified cardiac macrophages exposed to recombinant murine IL-10 expressed more osteopontin, a matricellular protein and multifunctional cytokine associated with cardiac fibrosis causing myofibroblast differentiation and activation (13). Fibroblasts are also the major targets of TGF-β secreted by macrophages under conditions of stress (67). In myocardial biopsies from HFpEF patients, cardiac macrophages increase gene expression of profibrotic TGF-β (59% compared to control) (68). *In vitro* experiments have demonstrated that TGF-β stimulates the transdifferentiation of myofibroblasts (69), enhances extracellular matrix (ECM) protein synthesis and increases integrin expression (70).

In a mouse model of cardiometabolic HFpEF, cardiac macrophages undergo a shift toward a proinflammatory phenotype. By secreting osteopontin and TNF-α, they bind to receptors on the surface of fibroblasts and activate downstream signaling pathways (62). This intercellular communication drives pathological activation of fibroblasts, leading to upregulation of genes such as Angiopoietin-like 4 (*Angptl4*) and collagen IV (*Col4a1*) (62). This promotes a distinct fibrotic process characterized by basement membrane remodeling, ultimately exacerbating cardiac diastolic dysfunction (62). ANGPTL4, as a secreted matricellular protein, plays multifaceted roles in HFpEF by regulating fibroblast activation, promoting microvascular rarefaction (71), and inhibiting lipoprotein lipase activity (72). In a murine HFpEF model, monocyte-derived CXCR4^+^ macrophages play an important role in fibrosis associated with HFpEF. They promote myofibroblast differentiation by significantly upregulating the expression of chemokine (C-X-C) motif ligand 3 (CXCL3), thereby contributing to fibrosis (73). In addition to cytokines, cardiac macrophages also secrete galectin-3, which promotes myocardial fibrosis by activating myofibroblasts (74). In HFpEF patients, plasma galectin-3 levels significantly increased from median baseline levels over 12 months (75).

### 3.3 Macrophages and endothelial cells

Cardiac microvascular endothelial cells produce paracrine factors such as nitric oxide (NO), prostacyclin, angiotensin II (Ang-II), and endothelin-1 (ET-1) to modulate cardiomyocyte contractility, growth and survival (76). Paulus et al. proposed a new paradigm for HFpEF development, suggesting that a co-morbidity-induced dysfunction of cardiac microvascular endothelium plays a central role in the development of cardiomyocyte hypertrophy and stiffness (77). He pointed out that in HFpEF, comorbidities lead to a systemic inflammatory state, which in turn triggers oxidative stress in the endothelium of the coronary microvasculature. This reduces myocardial NO bioavailability and leads to reduced protein kinase G (PKG) activity in cardiomyocytes, resulting in myocardial stiffness and hypertrophy (77). Research on interactions between endothelial cells and other cells has primarily focused on cardiac endothelial-cardiomyocyte interaction, with relatively fewer studies examining interactions between endothelial cells and immune cells. In a study, endothelial cells upregulated the expression of E-selectin, intercellular adhesion molecule-1 (ICAM-1) and vascular cell adhesion molecule (VCAM) in HFpEF patients and HFpEF rats (78). Expression of adhesion molecules favors myocardial infiltration of monocyte/macrophage. It has been proposed that the inflammatory state in HFpEF, resulting from increased pro-inflammatory molecules and the activation of immune cells, plays a key role in vascular remodeling and endothelial cell death (79).

## 4 Effects of the pathological environment in HFpEF on macrophages

Extracardiac comorbidities associated with HFpEF, such as hypertension, obesity, diabetes mellitus, chronic obstructive pulmonary disease (COPD), and chronic kidney disease, which promote systemic inflammation, have been implicated in HFpEF development (80), as shown in **Table 1**. These metabolic alterations triggered by comorbidities continuously fuel a pro-inflammatory state, known as metabolic inflammation (46). In metabolic disorders, the characteristic chronic inflammatory state culminates in HFpEF (81).

**Table 1:** Comorbidities of HFpEF and their pathological mechanisms.

Comorbidity	Key metabolic changes	Affected macrophage subsets	Major downstream consequences
Diabetes	Development of an inflammatory environment and lipid accumulation in the myocardium.	CCR2^+^ proinflammatory macrophages (enhanced myocardial infiltration and phenotypic polarization).	Fibrotic myocardial remodeling, myocardial injury, left ventricular dysfunction.
Obesity	Production of adipokines and chemokines.	Phenotypic shift from CCR2^-^ to CCR2^+^ macrophages.	Oxidative stress, microvascular damage, cardiomyocyte hypertrophy, and myocardial fibrosis.
Hypertension	Pro-inflammatory gene expression patterns (with IL-1β, in particular, being the primary driver of changes in gene expression)	Expansion and proliferation of TIMD4^high^MHC-II^low^ macrophage subpopulation.	Left ventricular diastolic dysfunction, concentric left ventricular hypertrophy.
COPD	Systemic inflammation, with elevated levels of inflammatory markers such as CRP, TNF-α, and IL-6.	Recruitment of CCR2^+^ macrophages.	Cardiovascular and pulmonary risk factors form a “cardiopulmonary continuum” through systemic inflammatory processes.

CCR2, C-C chemokine receptor 2; IL-1β, interleukin-1β; TIMD4, T-cell immunoglobulin and mucin domain-containing protein 4; MHC-II, major histocompatibility complex class II; COPD, chronic obstructive pulmonary disease; CRP, C-reactive protein; TNF-α, tumor necrosis factor-α; IL-6, interleukin-6.

**Figure 2:**
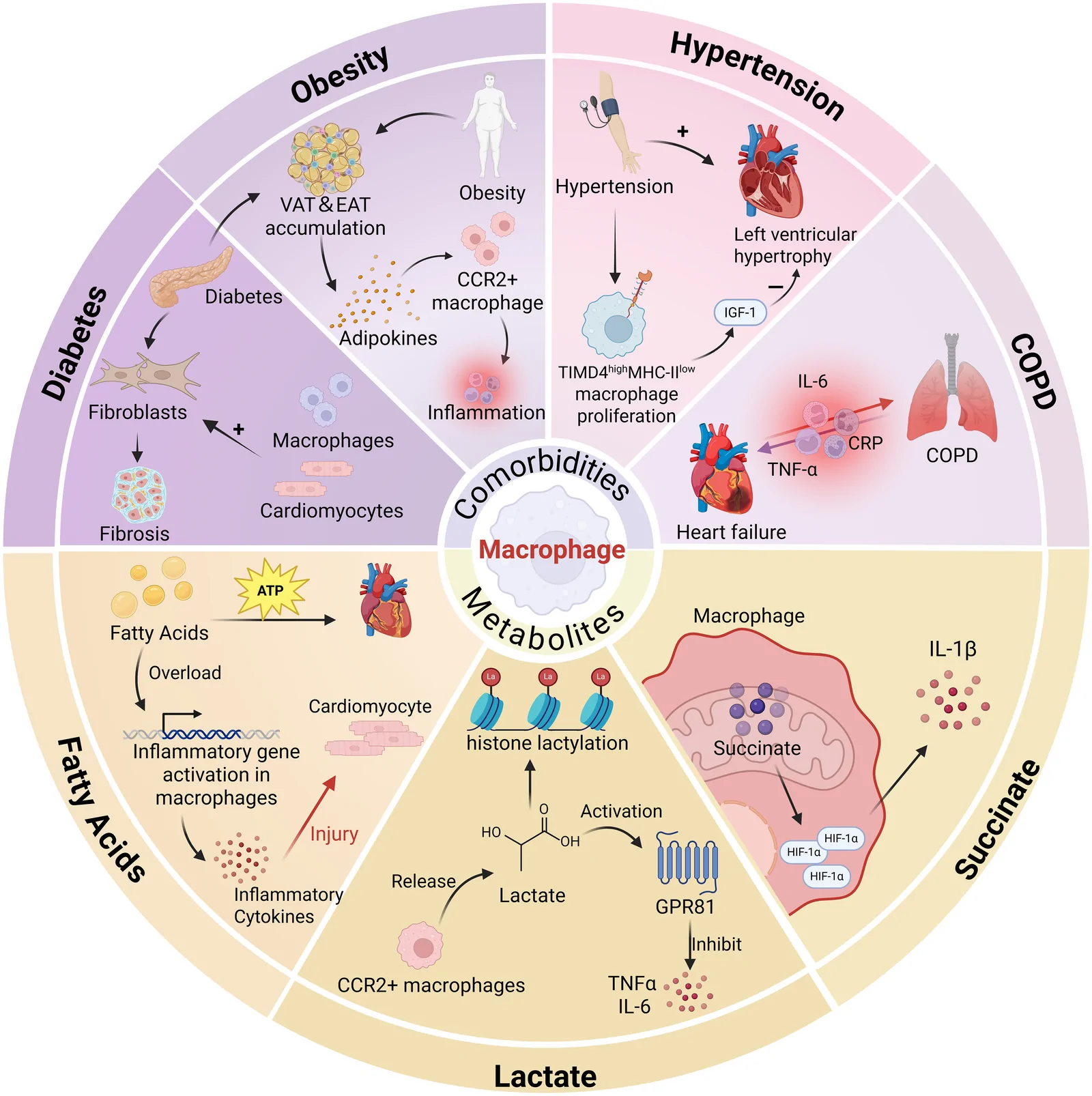
Systemic immunometabolic inflammation and cardiac immune metabolites: their effects on macrophages. Comorbidities such as diabetes, obesity, hypertension, and COPD can drive cardiac macrophage activation through inflammation and cytokines, thereby inducing myocardial injury, fibrosis, hypertrophy, and progression of HFpEF. Cardiac metabolites—such as fatty acids, lactate, and succinate—present in intracellular and surrounding extracellular spaces can alter the local cardiac microenvironment, linking macrophages to systemic metabolism through various mechanisms. VAT, visceral adipose tissue; EAT, epicardial adipose tissue; CCR2, C-C chemokine receptor 2; IGF-1, insulin-like growth factor 1; TIMD4, T-cell immunoglobulin and mucin domain-containing protein 4; MHC-II, major histocompatibility complex class II; COPD, chronic obstructive pulmonary disease; CRP, C-reactive protein; TNF-α, tumor necrosis factor-α; IL-1β, interleukin-1β; IL-6, interleukin-6; GPR81, G protein-coupled receptor 81; HIF-1α, hypoxia-inducible factor 1α.

The function of immune cells is driven by changes in intracellular metabolism and the availability of metabolites in the surrounding extracellular space (9). Alterations in metabolic programs within immune cells—particularly a rise in glycolysis accompanied by a fall in oxidative phosphorylation—promote immune cell activation and consequently amplify the inflammatory response (81). Cardiac metabolites alter the local cardiac environment. Specific cues regulating metabolic changes in the microenvironment can result in macrophage activation and polarization (82), as shown in **Table 2**. These metabolites, together with metabolic alterations induced by comorbidities, link macrophages to systemic metabolism (**Figure 2**).

**Table 2:** Metabolites and their signaling regulatory mechanisms.

Metabolites	Inflammatory characteristics	Major signaling molecules and regulatory pathways	Major effects and pathological findings	References
Fatty Acids	Pro-inflammatory when in excess	Lipid overload leads to the activation of pro-inflammatory genes and the release of pro-inflammatory cytokines in cardiac macrophages, affecting cardiomyocytes through the macrophage-cardiomyocyte regulatory axis.	Causes cardiomyocyte hypertrophy, fibrosis, and apoptosis.	(57)
Lactate	Anti-inflammatory/Context-dependent	GPR81 activation; induction of H3K18/H4K12 lactylation regulates macrophage function	Lactate released by CCR2^+^ macrophages activates GPR81, producing an anti-inflammatory effect; H3K18 lactylation suppresses inflammation, while H4K12 lactylation triggers inflammation.	(117–120)
Succinate	Dual Function	Intracellular succinate in macrophages stabilizes HIF-1α; extracellular succinate activates GPR91	Intracellular succinate stabilizes HIF-1α in macrophages, promoting the production of the pro-inflammatory cytokine IL-1β; in the HFpEF myocardium, extracellular succinate improves diastolic function and inhibits adverse remodeling by activating GPR91 signaling in cardiomyocytes.	(122, 124, 125)

CCR2, C-C chemokine receptor type 2; GPR81, G protein-coupled receptor 81; HIF-1α, hypoxia-inducible factor 1α; GPR91, G protein-coupled receptor 91; IL-1β, interleukin-1β; HFpEF, heart failure with preserved ejection fraction.

In addition to metabolism, the activation of the nervous system during heart failure can also affect macrophages and cardiac remodeling (83), which may play a key role in the pathogenesis of HFpEF. Here we explore the effects of HFpEF-related comorbidities, metabolites and the nervous system on the cardiac microenvironment and macrophages.

### 4.1 Systemic immunometabolic inflammation drives macrophage infiltration

#### 4.1.1 Diabetes

Diabetes is a main risk factor for diastolic dysfunction and heart failure with preserved ejection fraction (51), coexisting in nearly 45% of HFpEF cases (84). Fibrotic myocardial remodeling plays a pivotal role in the pathogenesis of diabetes-associated heart failure. Diabetes-related fibrosis is primarily mediated by activated cardiac fibroblasts, but macrophages, cardiomyocytes, and vascular cells may also promote the fibrotic process by modulating the phenotype and function of fibroblasts (85). However, it has not yet been directly verified whether the recruitment or activation of pro-fibrotic subpopulations within monocytes/macrophages mediates the fibrotic process in diabetic hearts (85). Diabetes, as an inflammatory disease, induces macrophage dysfunction through hyperglycemia, the accumulation of advanced glycation end products (AGEs), and oxidative stress. Activation of the AGE-RAGE signaling pathway promotes oxidative stress and further activates the NF-κB and NLRP3 inflammasome pathways (86), thereby enhancing the inflammatory response of macrophages and promoting increased expression of pro-inflammatory cytokines such as TNF-α, IL-1β, and IL-6, as well as various chemokines and vascular adhesion molecules. Diabetes also promotes the tissue infiltration of macrophages and their polarization toward a CCR2^+^ pro-inflammatory phenotype and increases the number of circulating monocytes and neutrophils (87). The influx of infiltrating inflammatory cells, particularly macrophages, has become a key factor in the development and progression of myocardial damage and left ventricular dysfunction in various cardiac conditions, including HFpEF and HFrEF (88). Myocardial lipid accumulation is associated with hyperglycemia and diabetes. Accumulated lipids induce the secretion of pro-inflammatory cytokines by myocardial and epicardial cells, attracting macrophages that impair myocardial and vascular function (89). This aspect will be discussed in detail in the section on obesity.

#### 4.1.2 Obesity

Among patients with established HFpEF, 70%-80% are obese (90). This relationship is more pronounced among women than among men (91). Obesity and metabolic stress trigger low-grade systemic inflammation alongside the dysregulation of inflammatory and immune responses. These altered responses are considered to be the pathophysiological basis of HFpEF (10). The accumulation of visceral adipose tissue (VAT) and epicardial adipose tissue (EAT) can induce both systemic and local inflammation. This inflammation leads to oxidative stress, microvascular injury, cardiomyocyte hypertrophy, and myocardial fibrosis, ultimately resulting in heart failure (10). The mechanism involves the accumulation of visceral and ectopic fat leading to the production of large quantities of adipokines and chemokines, which in turn serve as signaling molecules to recruit immune and inflammatory cells into the myocardium (92). VAT accumulation shows the strongest association with HFpEF development, hospitalization rates, and mortality (93).

In obese murine models, adipose tissue macrophages switch from a CCR2^-^ to a CCR2^+^ phenotype, thereby creating a more pro-inflammatory environment within adipose tissue (94). Neutrophils, other myeloid cells, and mast cells in adipose tissue promote tissue damage through elastase secretion, which also facilitates macrophage recruitment (95). Saturated fatty acids can activate the Toll-like receptor 4 (TLR4)/myeloid differentiation primary response 88 (MyD88)/NF-κB signaling pathway and the NLRP3 inflammasome (96), thereby leading to increased production of IL-1β, IL-6, and TNF-α. Simultaneously, the inflammatory state in VAT or EAT induces CCR2^+^ macrophage polarization and recruitment into the heart (97).

Obesity is often accompanied by an expansion of EAT (98). Through an unobstructed shared microcirculation, the epicardium and myocardium are closely linked. Consequently, when dysfunctional epicardial adipose tissue lies adjacent to the left ventricle, left ventricular distensibility becomes impaired, thereby giving rise to HFpEF (7). Additionally, the increased risk of HFpEF is associated with some extent with LV structural changes (wall thickness and intraventricular dimensions), poorer LV relaxation (longer isovolumic relaxation time), and increased filling pressures (larger left atrial diameter) (5). In addition, obesity induces cardiac fibrosis through a mechanism similar to pathological hypertrophy. This process is primarily driven by mechanical stress-induced signaling pathways in cardiac fibroblasts. However, macrophage-induced inflammation also serves as a contributor (99).

#### 4.1.3 Hypertension

Hypertension is a prevalent comorbidity in HFpEF (100). The first discernible manifestation in most hypertensive patients is LV diastolic dysfunction. Cardiac pressure overload induces concentric LV hypertrophy. When pressure overload persists, diastolic dysfunction progresses, the concentric remodeled LV decompensates, and HFpEF ensues (101). Hypertensive individuals exhibit pro-inflammatory expression patterns, with IL-1β being a prominent driver of observed gene expression changes (102). This may represent an immunological mechanism by which hypertension contributes to the development of HFpEF. Hypertension promotes macrophage activation primarily through neurohumoral mechanisms, particularly angiotensin II-mediated signaling (103). Angiotensin II activates angiotensin II type 1 (AT1) receptor-dependent pathways, including NADPH oxidase/reactive oxygen species (ROS), NF-κB, and MAPK signaling, resulting in increased expression of inflammatory cytokines and chemokines (103). In murine models of hypertension, cardiac resident macrophages have been demonstrated to increase numerically during both acute and chronic hypertension, and the TIMD4^high^MHC-II^low^ subpopulation is the only cardiac macrophage subset that persistently proliferates in hypertension. This subpopulation is an important source of IGF-1 in the heart, and hypertensive stress also elicits a cardiac resident macrophage response that includes production of IGF-1 (104). IGF-1 reduces the degree of LV hypertrophy, allowing the myocardium to withstand hypertensive stress (104). Thus, during hypertension, the proliferation of the TIMD4^high^MHC-II^low^ subpopulation enhances the core repair potential of cardiac macrophages.

#### 4.1.4 Chronic obstructive pulmonary disease

**Figure 3:**
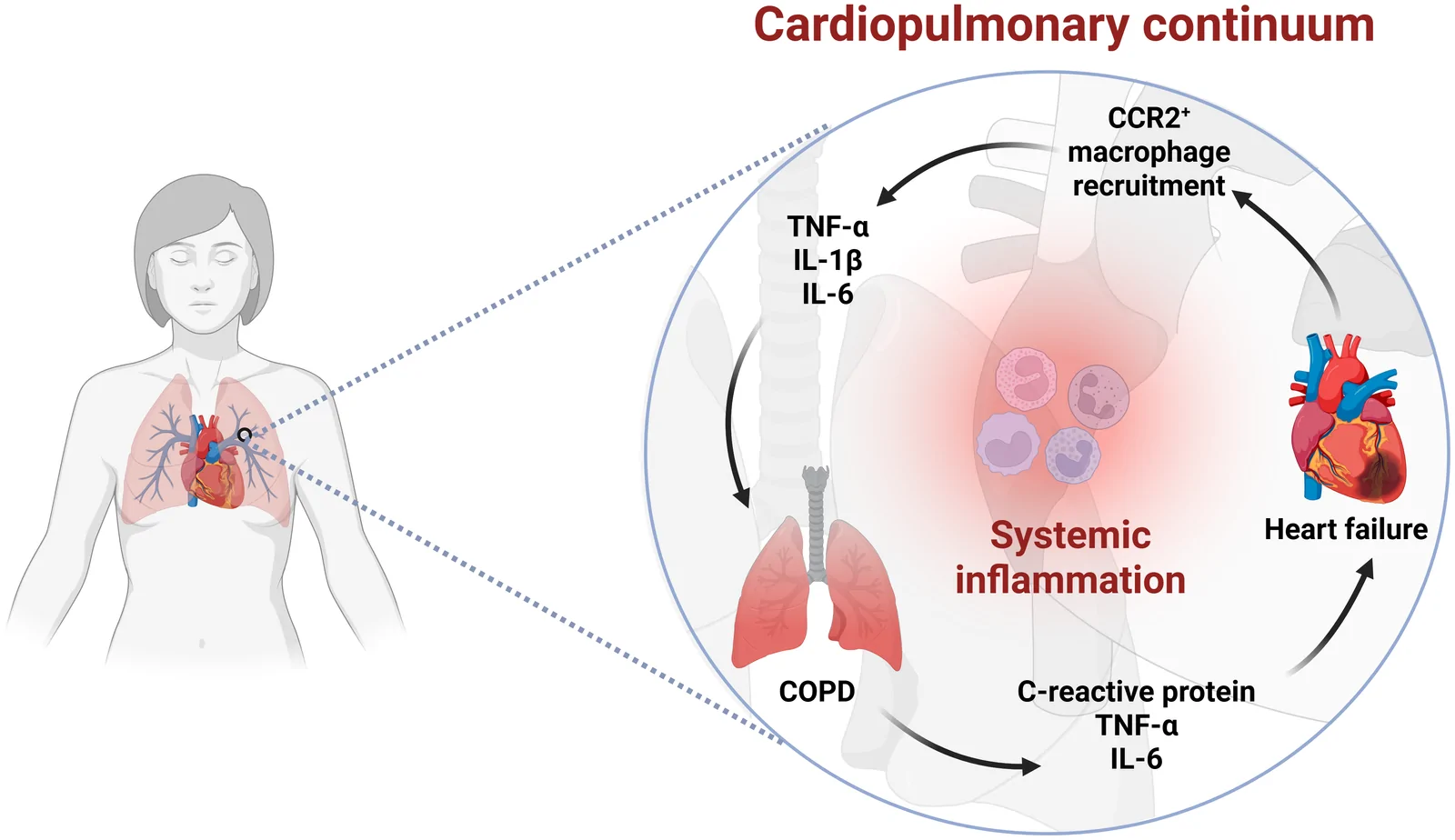
Low-grade systemic inflammation is considered a common pathophysiological link between COPD and heart failure. The low-grade systemic inflammation triggered by COPD leads to the release of CRP, TNF-α, and IL-6 into the circulation, inducing heart failure and recruiting CCR2^+^ macrophages. These macrophages subsequently release pro-inflammatory factors that act on the lungs, exacerbating tissue damage and airflow obstruction and thereby promoting the progression of COPD. This phenomenon demonstrates that cardiovascular and pulmonary diseases do not exist in isolation but are closely linked through shared inflammatory pathways, forming a “cardiopulmonary continuum”. CCR2, C-C chemokine receptor 2; TNF-α, tumor necrosis factor-α; IL-1β, interleukin-1β; IL-6, interleukin-6; COPD, chronic obstructive pulmonary disease.

Chronic obstructive pulmonary disease (COPD) is a common chronic inflammatory lung disease. It is characterized by persistent respiratory symptoms and fixed airflow obstruction resulting from airway inflammation (105). Approximately one-third of HF patients also have COPD, and the prevalence of COPD is higher among patients with HFpEF than among those with HFrEF (106). Low-grade systemic inflammation is considered a common pathophysiological link between COPD and cardiovascular diseases (107). The effect of COPD on macrophages in HFpEF is also achieved indirectly through the systemic inflammation it induces. However, the origin of systemic inflammation in COPD is not yet fully understood. Some studies suggest that certain common genetic or constitutional factors may predispose patients with COPD to both systemic and pulmonary inflammation. Pulmonary overinflation, tissue hypoxia, and changes in skeletal muscle and bone marrow are also thought to be associated with the induction of systemic inflammation (108). Previous studies have shown that levels of inflammatory markers such as C-reactive protein (CRP), TNF-α and IL-6 are elevated in COPD patients compared to healthy controls (108). CRP, TNF-α, and IL-6 enter the circulatory system, activating systemic inflammatory pathways, directly contributing to the pathogenesis of heart failure, and recruiting CCR2^+^ macrophages. When activated CCR2^+^ macrophages release inflammatory cytokines such as TNF-α, IL-1β, and IL-6 into the lungs, these cytokines can cause tissue damage, leading to emphysema, impairing the airways’ defensive and reparative functions, and ultimately resulting in small airway fibrosis and progressive, irreversible airflow obstruction (109). Therefore, cardiovascular and pulmonary risk factors form a “cardiopulmonary continuum” through shared systemic inflammatory processes, a pathophysiological relationship that revolves around a common inflammatory pathway (107), as shown in **Figure 3**.

### 4.2 Effects of cardiac immune metabolites on macrophages

#### 4.2.1 Fatty acids

As the heart’s primary fuel source, fatty acid oxidation accounts for 40–60% of cardiac ATP production (110). Heart failure is associated with significant changes in energy substrate utilization and overall metabolic distribution (110). In human HFpEF hearts, the myocardium relies more heavily on fatty acids as its primary source of fuel for ATP production, compensating for the reduced ATP derived from glucose oxidation by increasing the rate of fatty acid oxidation (111). CCR2^-^ macrophages utilize fatty acids as their primary energy source and exert anti-inflammatory effects through oxidative phosphorylation, thereby providing a sustained energy supply for tissue maintenance and repair (112). By contrast, CCR2^+^ macrophages predominantly shift from oxidative phosphorylation toward glycolysis, a rapid but inefficient metabolic pathway that provides a means to rapidly generate the building blocks required for cell proliferation and the energy needed to secrete inflammatory cytokines. This shift from oxidative phosphorylation to glycolysis sustains phagocytosis and cytokine secretion (12).

Enhanced fatty acid metabolism is a key driver of inflammatory activation. In a high-fat diet-induced dyslipidemia model, lipid overload directly impacts the activation of inflammatory genes in cardiac macrophages. Lipid accumulation markers are mainly expressed at a high level in Timd4^+^ resident macrophages, indicating that cardiac resident macrophages are the ones primarily subject to metabolic stress (57). Moreover, inflammatory cytokines secreted by macrophages under metabolic stress can influence pathological processes in cardiomyocytes, such as hypertrophy, fibrosis, and apoptosis, via the macrophage-to-cardiomyocyte regulatory axis, thereby contributing to the onset and progression of diastolic dysfunction (57).

#### 4.2.2 Lactate

During disease exacerbation episodes, when fatty acid availability becomes impaired, the heart can effectively use lactate as an alternative energy source (113). In addition, as a signaling molecule, extracellular lactate can directly engage immune cells and tissue parenchymal cells via lactate receptors, as well as influence metabolic pathways when it accumulates in the cytosol following uptake by lactate transporters (46). Activated CCR2^+^ macrophages upregulate glucose uptake and aerobic glycolysis, and release lactate as a by-product (114). G protein-coupled receptors (GPRs), as important cell surface receptors, play a key role in signal transduction in many cell types, including macrophages (115). GPR81 has been identified as the lactate receptor (116). *In vitro* macrophage experiments have shown that lactate promotes macrophage polarization toward an anti-inflammatory phenotype in a GPR81-dependent manner and inhibits CCR2^+^ macrophage polarization, while suppressing production of pro-inflammatory cytokines TNF-α and IL-6 (117, 118). This negative feedback loop facilitates the resolution of inflammation.

The effects of lactate on macrophages are also regulated by epigenetic modifications, such as histone lactylation. Lactate drives histone lactylation in macrophages, and lactylation at different sites can lead to substantial variations in their regulatory functions (119). H3K18 lactylation in macrophages regulates the anti-inflammatory and pro-angiogenic activities by promoting the transcription of reparative genes such as IL-10, vascular endothelial growth factor A (VEGF-A), and leucine-rich alpha-2-glycoprotein 1 (LRG1), thereby improving cardiac function and maintaining cardiac homeostasis (120). However, in a high-free fatty acid environment, lactate primarily exacerbates H4K12 lactylation. H4K12 accumulates in the promoter region of the key transcription factor hypoxia-inducible factor 1α (HIF-1α). The upregulation of HIF-1α expression mediates the release of pro-inflammatory factors, promotes inflammatory infiltration within the microenvironment, and thereby mediates the inflammatory response of macrophages. Research has found that inhibiting monocarboxylate transporter 4 (MCT4) in mice with high-fat-induced type 2 diabetes reverses H4K12 lactylation in cardiac macrophages and reduces cardiac inflammation (119). Although the lactylation-macrophage polarization-inflammation axis is regarded as a central mechanism in HFpEF progression, the exact sites of lactylation on macrophages remain inadequately characterized (121), and current evidence regarding the regulation of macrophage polarization by histone lactylation primarily comes from *in vitro* experiments and animal models. Therefore, future work should aim to elucidate the regulatory mechanisms underlying this process.

#### 4.2.3 Succinate

Succinate has been identified as a metabolite in innate immune signaling. As an endogenous inflammatory signal, intracellular succinate in CCR2^+^ macrophages induces the stable accumulation of the HIF-1α protein by inhibiting prolyl hydroxylase (PHD). HIF-1α, acting as a transcription factor for the pro-inflammatory cytokine IL-1β, binds to the promoter region of the *IL-1β* gene, thereby directly promoting the transcription and expression of IL-1β (122). Beyond its role as a metabolic intermediate, succinate also functions as a signaling molecule via its receptor GPR91, regulating glucose-lipid metabolism, inflammatory responses, and cellular energy homeostasis (123). GPR91 is expressed in multiple tissues and cell types, including cardiomyocytes and immune cells, but its functional effects upon activation are not always straightforward. Depending on the environment, it can induce either pro-inflammatory or anti-inflammatory responses (124). In an inflammatory environment, succinate released into the extracellular space binds to GPR91 on macrophages, stimulating CCR2^+^ macrophages to produce more IL-1β, thereby continuously amplifying pro-inflammatory signals and making it difficult for the inflammation to subside. In contrast, succinate levels are significantly reduced in HFpEF cardiomyocytes (125), and succinate exhibits protective potential, which contradicts its classical pro-inflammatory effects. Succinate ameliorates HFpEF by enhancing cardiac diastolic function and curbing adverse remodeling. This process is mediated through activation of GPR91 in cardiomyocytes. Although the immunomodulatory function of the succinate-GPR91 axis in cardiac macrophages is primarily based on studies using mouse models and cardiomyocyte cell lines, it is regarded as a critical regulator of cardiometabolic homeostasis and has become a potential therapeutic target for HFpEF (123).

### 4.3 Effects of nervous system on cardiac macrophages

Through complex neuroimmune interactions, the nervous system plays a key role in regulating the function of cardiac macrophages (126). Neurohumoral dysregulation, particularly excessive activation of the sympathetic nervous system (SNS) and renin–angiotensin–aldosterone system (RAAS), is commonly observed in symptomatic heart failure and contributes to disease progression and clinical manifestations (127). The heart-brain axis is a bidirectional communication network that integrates neural, endocrine, and immune signals and helps maintain cardiovascular homeostasis (128). In the heart-brain axis, sensory signals are transported from the heart to the brain via spinal afferents and the vagal nerve through the dorsal root ganglion, spinal cord or nodose ganglia. Patients with heart failure exhibit brain abnormalities and neurological disorders; the brain transmits signals through various pathways, prompting cardiac remodeling (129). This interconnection between the heart and the brain may represent a new therapeutic target.

The vagus nerve primarily modulates immune responses through cholinergic anti-inflammatory pathways, producing potent anti-inflammatory and anti-fibrotic effects in animals with HFpEF (130). The vagal afferent fibers detect peripheral inflammation and transmit action potentials from the periphery to the brainstem, thereby stimulating the descending vagus nerve to generate action potentials. Vagal efferent fibers regulate immune cells via cholinergic signaling; specifically, acetylcholine activates α7 nicotinic acetylcholine receptors (α7nAChRs) expressed on the surface of macrophages. Through intracellular signal transduction mediated by these receptors, the activity of NF-κB is inhibited, thereby suppressing the production of pro-inflammatory cytokines (131). Therefore, vagus nerve-mediated immune regulation is a neuroimmune regulatory mechanism involving neural signaling and the regulation of downstream cytokines, rather than direct neural innervation of immune cells.

In heart failure, excessive sympathetic nervous system activation also affects macrophages. Cardiac fibroblasts and nearly all immune cell types express beta-adrenergic receptors (βAR). Macrophages participate in the pathological processes of heart failure due to stimulation by norepinephrine and epinephrine released from sympathetic nerves (132). Patients with HFpEF exhibit elevated plasma renin activity, and activation of RAAS is considered the most relevant neurohormonal mechanism in HFpEF-associated fibrosis (83). While the crosstalk between the renin–angiotensin system and the immune system has been well documented, the roles of the adrenergic and natriuretic peptide systems in regulating immune responses are less clearly defined, with limited mechanistic evidence available to date (83). The interaction between neurohumoral pathways and the immune system in HFpEF remains to be further studied.

## 5 Therapeutic strategies targeting macrophages

The correlation between cardiac macrophages and the progression of HFpEF supports the development of macrophage-targeted therapies to promote the recovery of the failing heart (15). In this section, we review the most common and promising therapeutic strategies.

### 5.1 Drug therapy

Although broad anti-inflammatory therapies have not shown clinical benefits in patients with heart failure (133), the mechanism of action of many drugs for the treatment of HFpEF has been proved to be closely related to macrophages and immunity, as shown in **Table 3**. Sodium-glucose cotransporter 2 inhibitors (SGLT2i) are considered a promising treatment for HFpEF. They reduce the accumulation of CCR2^+^ macrophages, alleviate inflammation, and improve the metabolic abnormalities associated with HFpEF (134–137). Statins can improve survival rates in patients with HFpEF (138). They may exert an additional immunomodulatory effect by limiting the accumulation of cholesterol within macrophages and inhibiting pro-inflammatory macrophage polarization (77). Colchicine has demonstrated anti-inflammatory effects in animals with HFpEF, reducing cardiac inflammation and the recruitment of CCR2^+^ macrophages (139); however, its long-term efficacy and safety in HFpEF require further validation. Other therapeutic strategies, including rapamycin (140), traditional Chinese medicine interventions (141), and vitamin B6 supplementation (142), have demonstrated protective effects in HFpEF models by modulating macrophage activation, metabolism, or polarization. Nevertheless, most of these strategies remain limited by insufficient clinical evidence, uncertainty regarding the optimal timing of treatment, and potential off-target effects.

**Table 3:** Drugs with mechanisms of action related to macrophages.

Drug	Mechanism of action	Therapeutic effect	References
Sodium–glucose cotransporter 2 inhibitor (SGLT2i)	SGLT2i reduce the accumulation of CCR2^+^ macrophages and increase the number of CCR2^-^ macrophages, thereby reducing inflammation. SGLT2i can also suppress weight gain and reduce obesity-induced inflammation by enhancing fat utilization and browning.	SGLT2i should be considered foundational therapy in all patients with heart failure. It improves cardiac function and clinical outcomes in patients with HFpEF.	(57, 134–137)
Statins	By reducing lipid levels, statins prevent the buildup of cholesterol esters in macrophages, a process that would otherwise drive polarization toward a pro-inflammatory phenotype.	Statins improve outcomes in patients with HFpEF and reduce mortality.	(77, 138)
Colchicine	Colchicine significantly lowers cardiac inflammation and reduces CCR2^+^ macrophage and neutrophil infiltration.	Colchicine treatment alleviates cardiac dysfunction and improves cardiac function, activity tolerance, and ventricular remodeling.	(139)
Rapamycin	Rapamycin suppresses inflammation by inhibiting the mechanistic target of rapamycin (mTOR), which enhances CCR2^+^ macrophage inflammation through glycolysis. At the same time, it can inhibit pro-fibrotic CCR2^+^ macrophages in HFpEF.	Rapamycin is beneficial to the diastolic function and myocardial stiffness of HFpEF.	(140)
Traditional Chinese Medicine	Resveratrol and other Traditional Chinese Medicine modulate cardiac inflammation in HFpEF mice by promoting the transition from CCR2^+^ macrophages to CCR2^-^ macrophages.	Traditional Chinese Medicine interventions have been shown to exert a protective action against HFpEF-induced adverse cardiac remodeling.	(141, 160)
Vitamin B-6	Vitamin B-6 suppresses inflammation by inhibiting macrophage activation, as evidenced by reduced production of TNF‐α and IL‐1β *in vivo*.	Vitamin B-6 prevents HFpEF in the mouse model.	(142)

SGLT2i, sodium-glucose cotransporter 2 inhibitors; HFpEF, heart failure with preserved ejection fraction; CCR2, C-C motif chemokine receptor 2; mTOR, mechanistic target of rapamycin; TNF-α, tumor necrosis factor-α; IL-1β, interleukin-1β.

### 5.2 Potential targets

In recent years, macrophage-targeted therapy has garnered increasing attention in the field of cardiac immunology (143). Given the critical role of cardiac macrophages in HFpEF pathogenesis, several potential therapeutic targets have been proposed, including macrophage phenotype regulation, chemokine signaling (such as CCR2 and CXCR4), microRNAs, inflammatory cytokines, inflammasome pathways, and metabolic regulators, as shown in **Table 4**. However, the number of these studies on potential targets is limited, and most of them are in early exploration or preclinical research.

**Table 4:** Macrophage-related therapeutic targets.

Target		Potential mechanisms	References
CCR2^-^ macrophages	TREM2	TREM2 upregulation in cardiac macrophages prevents HFpEF by promoting anti-inflammatory and pro-angiogenic programs.	(37, 161)
CCR2^+^ macrophages	CCR2	CCR2 antagonist blocks the accumulation of infiltrating macrophages in the heart and alleviates late pathological left ventricular remodeling, dysfunction and cardiac fibrosis.	(157)
	CXCR4	In HFpEF heart tissue, the absence of CXCR4 suppresses both macrophage infiltration and the inflammatory response, resulting in reduced cardiac fibrosis and improved diastolic function. Meanwhile, CXCR4 augments the pro-inflammatory state in macrophages by inhibiting PPARγ activity.	(73)
MicroRNAs (miRNAs)	MiR-21	MiR-21 in macrophages is required for their polarization toward the CCR2^+^ phenotype. In the setting of left ventricular pressure overload, genetic deletion of miR-21 in macrophages confers protection against interstitial fibrosis and cardiac dysfunction.	(162)
	MiR-223-3pMiR-486-3p	MiR-223-3p and miR-486-3p are identified as key drivers of macrophage polarization toward a pro-inflammatory phenotype, making them potential therapeutic targets for heart failure.	(163)
Cytokines	IL-1	IL-1 blockade significantly alleviates the systemic inflammatory response in HFpEF patients.	(164)
Dendritic cell-specific intercellular adhesion molecule-3-grabbing nonintegrin (DC-SIGN)		Treatment with DC-SIGN ligand 1 (DCSL1) mitigates the changes in inflammation, cardiac function, and fibrosis in female mice by reducing the number of fibroblast-promoting macrophages. However, the treatment is not effective in male mice, which may be due to differences in the inflammatory cell profiles of the hearts between male and female mice.	(165, 166)
NLRP3 inflammasome		The NLRP3 inflammasome inhibitor MCC950 attenuates the circulating pro-inflammatory cytokine IL-18, and lowers macrophage infiltration into cardiac tissue.	(167)
Nicotinamide N-methyltransferase (NNMT)		In the context of HFpEF, the NNMT inhibitor AMO-NAM shows potential to lower systemic inflammation as well as macrophage infiltration into cardiac tissue and visceral adipose tissue.	(168)

TREM2, triggering receptor expressed on myeloid cells 2; CXCR4, C-X-C motif chemokine receptor 4; PPARγ, peroxisome proliferator-activated receptor γ; IL-1, interleukin-1; NLRP3, NOD-like receptor family pyrin domain-containing protein 3; IL-18, interleukin-18; NNMT, nicotinamide N-methyltransferase.

### 5.3 Short-chain fatty acids

Short-chain fatty acids (SCFAs) may have great potential in preventing or managing HFpEF. They have been shown to decrease circulating long-chain fatty acids (LCFAs) and glucose levels, potentially counteracting the development of cardiac steatosis and glucotoxicity (144). Concurrently, SCFAs possess anti-inflammatory and immunomodulatory effects. The primary mechanism by which they recruit macrophages is epigenetic. SCFAs, particularly butyrate, act as histone deacetylase (HDAC) inhibitors. This epigenetic regulation has been found to interfere with the maturation process of dendritic cells, suppress CCR2^+^ macrophage differentiation, and reduce the expression of restricted membrane antigens and the phagocytic capacity of cells (145). Therefore, raising the levels of SCFAs could be an attractive therapeutic strategy for HFpEF. Evidence indicates that high-fiber diets enhance gut microbial diversity and promote bacteria capable of producing SCFAs. This process improves cardiac function while mitigating pressure overload-induced cardiac fibrosis and hypertrophy (146). In fact, an inverse association between dietary fiber intake and cardiovascular disease risk has been well documented (147), making a high-fiber diet a recommended nutritional approach for patients with heart failure.

### 5.4 Transcutaneous vagus nerve stimulation

Transcutaneous vagus nerve stimulation (tVNS) has emerged as a highly promising therapeutic strategy for various cardiovascular diseases, including heart failure with preserved ejection fraction (148), atrial fibrillation, and autonomic dysfunction. The vagus nerve modulates immune responses by activating the neuroimmune cholinergic anti-inflammatory pathway. This pathway exerts strong anti-inflammatory and anti-fibrotic effects in both animals and humans. Furthermore, it significantly improves the HFpEF phenotype in a high-salt diet rat model (130). Secreted Phosphoprotein 1 (Spp1/osteopontin) activates myofibroblasts by binding to its receptor on fibroblasts, promoting extracellular matrix production and fibrosis. tVNS reduces Spp1 expression in CCR2^+^ macrophages, thereby decreasing myocardial fibrosis. Concurrently, tVNS upregulates the expression of the repair gene IGF1 in TLF^+^/MHC-II^+^ cardiac macrophages, promoting myocardial repair and adaptation (130). These findings reveal the neural-immune interaction mechanisms underlying HFpEF and provide a mechanistic basis for non-invasive therapeutic strategies.

### 5.5 Cardiosphere-derived cells

Cardiosphere-derived cells (CDCs) are heart-derived cell products with antifibrotic, anti-inflammatory and angiogenic properties (149). CDC therapy has been shown to reverse the functional abnormalities of HFpEF and improve survival in a rat model of HFpEF (150). The levels of pro-inflammatory and pro-fibrotic cytokines were reduced in CDC-treated HFpEF rats, and cardiac macrophages (CD68^+^ macrophages) were reduced by half (150). Distinctive changes in macrophage gene expression and function have been shown to underlie the cardioprotective effects of CDCs. Al Shaimaa Hasan et al. demonstrated that CDC-conditioned medium polarized macrophages from the CCR2^+^ phenotype into the CCR2^-^ phenotype *in vitro* (151). However, Geoffrey de Couto et al. demonstrated through a rat myocardial infarction model that CDCs polarize macrophages away from the CCR2^+^ phenotype, but not toward a CCR2^-^ state, instead driving them toward a distinctive cardioprotective phenotype (152). Overall, CDC treatment has become a very promising approach for the treatment of HFpEF by restoring the diastolic function of the heart and alleviating fibrosis and inflammation (153).

## 6 Perspectives

As a complex syndrome, HFpEF is closely related to systemic low-grade inflammation and metabolic events. Recent studies have shown that cardiac macrophage-mediated immunometabolic regulation plays a key role in the whole process of HFpEF. It is important to note that macrophages play distinct roles in healthy and diseased hearts due to their heterogeneity. This review synthesizes emerging evidence on macrophage heterogeneity, intercellular communication, and metabolic reprogramming, proposing a comprehensive immunometabolic framework for the pathogenesis of HFpEF. HFpEF is driven not only by static macrophage phenotypes but also by a dynamic metabolic inflammation axis. This axis is modulated by extracardiac comorbidities as well as intracellular metabolites, including fatty acids, lactate, and succinate, which serve as key regulatory checkpoints. In recent years, progress in characterizing cardiac macrophages and their involvement in HFpEF has been considerable, which has led to the development of macrophage-oriented therapeutic strategies. However, macrophage-targeted therapies remain at an early stage. Further investigation is needed to fully ascertain their clinical efficacy and safety, thereby better informing novel treatment approaches and promoting favorable clinical outcomes.

### 6.1 Unresolved questions and future research directions

Although research has identified functions of cardiac macrophages in the context of heart failure, several questions remain to be addressed. For example, the origin and phenotype of resident macrophages are not yet fully understood. Furthermore, it remains unclear whether macrophage function is a cause or a consequence of disease progression in humans (31).

Secondly, the crosstalk between macrophages and cardiomyocytes, fibroblasts, and endothelial cells, as well as the effects of comorbidities and risk factors on macrophage heterogeneity and function, needs to be further explored to clarify the role of macrophages in HFpEF and provide guidance for therapeutic strategies (15).

Epigenetic regulation of macrophage immune metabolism in HFpEF warrants further attention. Although macrophage polarization is regulated by histone lactylation, specific histone modification sites, target genes, and regulatory networks in cardiac macrophages remain unclear. Elucidating these mechanisms will provide new insights for targeted therapies.

### 6.2 Challenges in translational research

Macrophage-targeted therapies face significant translational challenges. One of the challenges lies in the fact that, due to their inherent heterogeneity, it is essential to selectively target either CCR2^+^ macrophages or CCR2^-^ macrophages, while further distinguishing between subpopulations and their functions within mixed populations. In HFpEF, the inflammatory environment may cause cardiac macrophages to shift from a reparative phenotype to a pro-inflammatory phenotype. Consequently, the same intervention may yield opposite outcomes depending on the timing of administration and the target macrophage subset (53).

It is also challenging to achieve precise and targeted regulation of macrophages by drugs. New interventions are needed to enhance the selectivity and durability of drugs and avoid nonspecific off-target effects, thereby achieving safe and long-lasting therapeutic outcomes (53).

Investigations using animal models have advanced understanding of the complexity and plasticity exhibited by resident and recruited cardiac macrophages in both homeostasis and chronic heart failure (33). However, their predictive value is still low (154), and it is unclear whether these models can mimic human disease. In the future, it will be necessary to further develop animal models that closely mimic the clinical manifestations of the disease. Recently, human induced pluripotent stem cell (iPSC)-derived cardiac organoids have emerged as promising platforms for modeling human HFpEF by incorporating human-specific cardiac cellular responses and disease-associated phenotypes (155). These human-based models may provide new opportunities to investigate disease mechanisms, evaluate macrophage-mediated cardiac remodeling, and improve the translational potential of therapeutic interventions.

Another major challenge in translational research on HFpEF is the significant impact of comorbidities on disease heterogeneity and immune dysregulation. Unlike HFrEF, HFpEF is closely associated with systemic metabolic and inflammatory conditions, including obesity, diabetes, hypertension, chronic kidney disease, and chronic obstructive pulmonary disease. Therefore, future treatment strategies should take into account disease-related comorbidities rather than adopting a one-size-fits-all approach (156).

### 6.3 Future therapeutic directions

Promising therapeutic directions include (1): Methods for inhibiting CCR2 using antagonists or RNA-based technologies (157); (2) Secretome inhibition in CCR2^+^ macrophages, such as inhibition of macrophage-derived IL-1, IL-6 and TNFα (158); (3) Baroreflex activation therapy (BAT) and related device-based approaches may relieve pressure overload through implantable programmable pulse generator devices that restrain sympathetic nervous system activity and augment parasympathetic nervous system influences, thereby ameliorating HFpEF (159); (4) Using butyrate or high-fiber diets to modulate gut microbiota and enhance macrophage anti-inflammatory effects.

Cardiac macrophages are central to HFpEF immunometabolic regulation, and targeting their subsets, pathways, and regulatory molecules holds great therapeutic promise. Future research should address mechanistic gaps, overcome translational challenges, and develop precise therapies. Advances in research techniques and mechanistic understanding are expected to make macrophage-mediated immunometabolic regulation a key breakthrough in improving HFpEF diagnosis, treatment, and patient outcomes.
